# 2423: Social determinants of health and comorbidity in individuals with type 2 diabetes at HealthStreet, a community engagement initiative

**DOI:** 10.1017/cts.2017.274

**Published:** 2018-05-10

**Authors:** Scott Cohen, Jasmine Mack, Catherine Striley, Linda Cottler

## Abstract

OBJECTIVES/SPECIFIC AIMS: Research on social determinants of health (SDHs) in type 2 diabetes have largely examined disease etiology rather than severity. To find factors associated with complications, we investigated socio-demographics, healthcare access, and healthcare utilization in individuals with type 2 diabetes with respect to related comorbidity. METHODS/STUDY POPULATION: Community health workers assessed 8494 participants for type 2 diabetes (n=939; 11%) through HealthStreet, a community-engagement model implemented in North Central Florida. Comorbidities were defined as neuropathy, retinopathy, high cholesterol, hypertension, and kidney failure. We conducted multivariate analyses to test the association of socio-demographic factors and comorbidity status. RESULTS/ANTICIPATED RESULTS: Of 939 members with type 2 diabetes, 164 (17%), 272 (29%), 370 (39%), and 133 (14%) reported having 0, 1, 2, and 3+ comorbidities, respectively. There is a smaller proportion of African-Americans reporting 3+ comorbidities compared with other comorbidity groups (*p*=0.003). Those with more comorbidity are less employed (*p*<0.0001) and are more likely to have Medicare/Medicaid (*p*=0.03) than those without comorbidity. Those with no comorbidity are more likely to be uninsured compared to those with comorbidity (*p*=0.0297). Adjusting for age, race, gender, and BMI, those that have at least 1 comorbidity are 1.4 times more likely to be food insecure (*p*=0.004) and are 1.9 times more likely to have seen a doctor in the past 12 months (*p*=0.002) compared to those without comorbidity. DISCUSSION/SIGNIFICANCE OF IMPACT: Although there is complexity among the relationships between SDHs and diabetic comorbidity, results suggest significant sociodemographic and healthcare-related disparities among individuals living with type 2 diabetes. Members with more comorbidity utilize healthcare, but are more likely to be food insecure among other factors. Those with no comorbidity are least likely to see a physician, which could imply a gap in the care continuum. This analysis gives insight into the importance of efficient diabetes management, focused on disparities in economic stability and healthcare access and utilization.

